# D-sorbitol can keep the viscosity of dispersive ophthalmic viscosurgical device at room temperature for long term

**DOI:** 10.1038/s41598-019-53390-0

**Published:** 2019-11-14

**Authors:** Eiji Nogami, Ippei Watanabe, Hirotaka Hoshi, Masakazu Kasahara, Naoto Honda, Miwako Sato, Kiyoshi Suzuki

**Affiliations:** 1 Kurihama Plant, Seikagaku Corporation, 9-3-1, Kurihama, Yokosuka, Kanagawa 239-0831 Japan; 2 Medical Science Liaison Unit, Seikagaku Corporation, 1-6-1 Marunouchi, Chiyoda-ku Tokyo, 100-0005 Japan; 3 Central Research Laboratories, Seikagaku Corporation, 3-1253 Tateno, Higashiyamato Tokyo, 207-0021 Japan; 4 Takahagi Plant, Seikagaku Corporation, 258-5 Aza-Matsukubo, Oaza-Akahama, Takahagi Ibaraki, 318-0001 Japan

**Keywords:** Molecular medicine, Drug discovery and development

## Abstract

The combination of 3% sodium hyaluronate (HA) and 4% sodium chondroitin sulfate (CS) is used as a dispersive ophthalmic viscosurgical device (OVD) during cataract surgery. For most OVDs containing HA, storage at 2–8 °C is recommended to preserve product characteristics. In order to develop a dispersive OVD that can be stored at room temperature, in this study, we searched additives which can stably maintain the viscosity, a key parameter of OVD, under preservation stability testing at 60 °C. The addition of D-sorbitol to a combination OVD, 3% HA and 4% CS, suppressed the reduction in viscosity compared with other OVDs with or without additives. The addition of D-sorbitol was also effective in improving the residual viscosity of the combination OVD after thermal treatment and light irradiation. Moreover, the OVD containing D-sorbitol can be stored at 25 °C with stably maintaining the initial viscosity for at least 24 months. In conclusion, the new dispersive OVD, 3% HA, 4% CS, and 0.5% D-sorbitol, can be stored at room temperature instead of under cold conditions and may represent an attractive option for clinical use because it is not necessary to bring the product to room temperature prior to use.

## Introduction

Sodium hyaluronate (HA) and sodium chondroitin sulfate (CS) are glycosaminoglycans (GAGs), which are widely distributed in the extracellular matrix and on the cell surface of animal tissues^1,2^. HA in particular represents a major lubricating component of the extracellular matrix and has been suggested to enhance sliding between adjacent tissue layers^3^.

Unlike most tissues in the eye, the cornea does not contain any blood vessels for nourishment or protection. When corneal endothelium is damaged by mechanical injury or bubble formation during phacoemulsification and aspiration (PEA), the endothelial cells cannot regenerate^4,5^. In such circumstances, ophthalmic viscosurgical devices (OVDs) are indispensable tools for the protection of corneal tissues. Various kinds of HA-based products have been commercialized as OVDs for use in cataract surgery, intraocular lens implantation, and penetrating keratoplasty^6^.

OVD products are classified into two major types, cohesive and dispersive, based on the physical properties of their viscoelastic materials^7^. The cohesive OVDs, e.g., Healon^®^ (Abbott Medical Optics Inc., CA, USA) and OPEGAN Hi^®^ (Santen Pharmaceutical Co., Ltd., Osaka, Japan), containing 1% HA with an average mass of >2000 kDa, show high cohesive properties^8–10^. These higher-viscosity cohesive agents are used to protect intraocular tissues from invasive surgical instruments and intraocular lenses during surgery by maintaining a deep anterior chamber^10^. However, this type of OVDs tends to easily flow out of the eye during PEA.

The dispersive OVDs, such as Viscoat^®^ (Alcon Inc., Hünenberg, Switzerland), a combination OVD consisting of 3% HA and 4% CS, are strongly retained in the anterior chamber during anterior segment surgery because of their adhesive nature in comparison with cohesive OVDs^11^, and thus display excellent corneal endothelial protection during PEA^12^. The disadvantages of dispersive OVDs are that it is insufficient to maintain space and is relatively difficult to remove. Additionally, the residual OVD may cause increase of postoperative intraocular pressure^13^. Therefore, if a dispersive OVD is used during cataract surgery, it is recommended to use cohesive OVD at the same time.

Both types of OVDs containing GAGs are relatively unstable against light irradiation and thermal treatment. More specifically, such treatments accelerate the depolymerization of GAGs, which reduces OVD viscosity^14,15^. Therefore, to maintain the important viscosity as rheological properties of these OVD products need to be stored in a refrigerator away from light until use. In consequence, prior to use, the temperature of such OVDs needs to be brought back to room temperature (preferably 15 °C to 25 °C), which may take 20 to 40 min^16^.

Generally, physico-chemical degradation of HA can occur in one of the five following manners: acid or alkaline hydrolysis, ultrasonic, thermal, oxidation, and photo-degradation^17^. Light irradiation and thermal treatment can accelerate the generation of free radicals, which can elevate oxidative stress and damage GAGs, leading to their depolymerization. For achieving long-term storage of the OVD products at room temperature, formation of oxygen-derived free radicals, which facilitate oxidation including photosensitized oxidation, should be suppressed as far as possible^18,19^.

In the present study, we evaluated the effect of various additives on the rheological properties of OVD products. Based on our observations, we have developed a new dispersive-type OVD, Shellgan^®^ (Santen Pharmaceutical Co., Ltd., Osaka, Japan) containing 3% HA, 4% CS, and 0.5% D-sorbitol. The addition of D-sorbitol to conventional OVD helped maintain stable OVD viscosity compared with additive-free conventional OVD. Subsequently, the D-sorbitol-containing OVD can be stored at room temperature for at least 24 months without reducing its viscosity.

## Results

### Selection of the additive by preservation stability testing at 60 °C

We evaluated the effect of various additives, i.e., D-sorbitol, glycine (Gly), L-glutamate (Glu), monosodium L-glutamate (MSG), L-methionine (Met), D-glucose (Glc), maltose monohydrate, xylitol, and D-α-tocopherol, on the viscosity of the basal OVD (Sample *a*) as shown in Table 1.

**Table 1:** Composition of samples.

Sample	GAG		Additive
	(4%)	(3%)	(0.5%)
*a*	CS	HA	—
*b*	CS	HA	D-sorbitol
*c*	CS	HA	glycine
*d*	CS	HA	L-glutamate
*e*	CS	HA	monosodium L-glutamate
*f*	CS	HA	L-methionine
*g*	CS	HA	D-glucose
*h*	CS	HA	maltose monohydrate
*i*	CS	HA	xylitol
*j*	CS	HA	D-α-tocopherol
*k*	CS	HA	1.0% D-sorbitol

Table 2 shows the time-dependent change in viscosity of the OVD samples stored at 60 °C. Initial viscosity of each sample with additive showed almost the same value as that of the basal OVD (sample *a*). The viscosity of the basal OVD reduced in a time-dependent manner. The viscosity measured after 14 days was 33.2% of the initial value. Upon adding sugar alcohols to the basal OVD (samples *b* and *i*), the progression of their viscosity reduction was decelerated. Particularly, the OVD containing 0.5% D-sorbitol showed viscosity of 25.53 Pa·s even after storage at 60 °C for 14 days. By adding D-sorbitol (sample *b*), the residual viscosity was improved, with the final viscosity being up to 50.5% of the initial value. Addition of Met, Glc, maltose, or D-α-tocophenol had no effect on viscosity (samples *f*, *g*, *h*, and *j*) and addition of amino acids, except Met, strongly accelerated the viscosity reduction (samples *c*, *d*, and *e*).

**Table 2:** Time-course of viscosity in the preservation stability test at 60 °C.

Sample	Additive	Viscosity (Pa·s)				
		0	Day 3	Day 7	Day 10	Day 14
*a*	—	50.54	41.64	27.56	20.61	16.76
*b*	D-sorbitol	—	44.83	36.10	28.91	25.53
*c*	glycine	—	8.49	1.07	0.05	0.00
*d*	L-glutamate	—	10.43	0.99	0.08	0.00
*e*	monosodium L-glutamate	—	26.77	8.64	4.37	2.51
*f*	L-methionine	—	40.44	29.61	23.85	18.73
*g*	D-glucose	—	38.92	22.73	15.59	9.37
*h*	maltose monohydrate	—	43.70	29.77	22.88	17.31
*i*	xylitol	—	44.66	32.49	26.69	19.33
*j*	D-α-tocopherol	—	38.22	25.68	19.99	15.83

Further, adding D-sorbitol did not accelerate browning of the basal OVD, judged by visual observation. Based on these results, D-sorbitol was selected as the best additive among the ones tested, and further detail examinations were performed using the new composition containing D-sorbitol.

### Effect of D-sorbitol concentration on the stability of the basal OVD against heat and light

We evaluated the effect of various concentrations of D-sorbitol on the viscosity reduction of basal OVD induced by thermal treatment or light irradiation.

Table 3 shows that the residual viscosity of the basal OVD was 49.05% of the initial value after autoclaving and 85.18% after photostability testing (sample *a*). Light shielding by using an aluminum foil was effective in preserving the viscosities of all the samples in the photostability testing, although a slight reduction in viscosity was observed in every OVD. The addition of D-sorbitol improved the photostability of the OVD as much as light-shielding. No conspicuous difference in viscosity loss was observed between the 2 D-sorbitol concentrations tested. In thermal stability testing, similar to photostability testing, viscosity loss was observed upon addition of D-sorbitol, but no difference in effect was observed for different D-sorbitol concentrations. Therefore, further studies were performed using OVD containing 0.5% D-sorbitol.

**Table 3:** Stability of the OVD containing D-sorbitol against thermal treatment and light irradiation. Residual viscosity (%) was calculated by the following numerical formula: [Viscosity of sample at the post-thermal treatment or light irradiation (Pa·s)]/[Initial viscosity of each sample (Pa·s)] × 100. The values represent the mean (*n* = 2).

Sample	D-sorbitol (%)	Residual viscosity (%)		
		Thermal treatment^†^	Light irradiation^‡^	
			Non-shielding	Shielding
*a*	0.0	49.05	85.18	89.00
*b*	0.5	65.71	90.53	95.03
*k*	1.0	67.97	92.04	93.80

^†^Autoclaving at 121 °C for 5 min.

^‡^2000 lx h^−1^, 25 days, 25 °C.

### Long term preservation stability of the new composition at 5 °C or 25 °C

**Figure 1:**
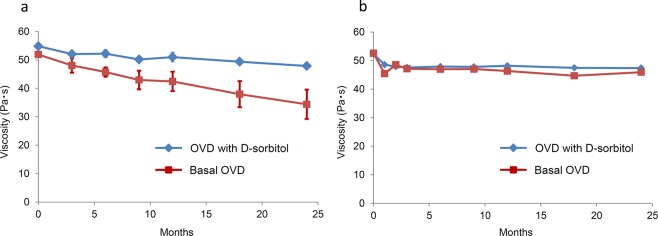
Long-term preservation stability of OVDs at 25 °C and 5 °C. (**a**) Time-course of viscosity of OVDs at 25 °C. The values represent the mean ± standard deviations (*n* = 3). (**b**) Time-course of viscosity of OVDs at 5 °C. The values represent the mean (*n* = 2).

We evaluated whether addition of D-sorbitol could improve the long-term stability of basal OVD, and the results are shown in Fig. 1. Viscosity reduction of both OVDs was negligible, independent of D-sorbitol addition, over 24 months of preservation testing at 5 °C; their residual viscosity after 24 months was over 87%. When the OVDs were stored at 25 °C, the viscosity of the basal OVD reduced in a time-dependent manner, the residual viscosity at the end of the test became less than 67%, and the value dropped below 35 Pa·s. By adding D-sorbitol, the final viscosity of the basal OVD stored at 25 °C was maintained between 55 and 47 Pa·s for 24 months. The residual viscosity of the new composition at the end-point was over 87%, indicating that addition of D-sorbitol improved the long-term stability of the basal OVD enough for storage at room temperature, as effectively as low-temperature storage.

## Discussion

Non-enzymatic reactions that degrade HA are classified into five types: acidic and alkaline hydrolysis, ultrasonic degradation, thermal degradation, degradation by oxidants, and miscellaneous degradations like photodegradation^17^. Particularly, we hypothesized that the viscosity reduction of OVD products during preservation was caused by the degradation of HA by hydroxyl radicals, because Hokputsaa *et al*. demonstrated that the values for the intrinsic viscosity and molecular weight of HA decreased due to hydroxyl radicals, depending on their amount generated^20^.

**Figure 2:**
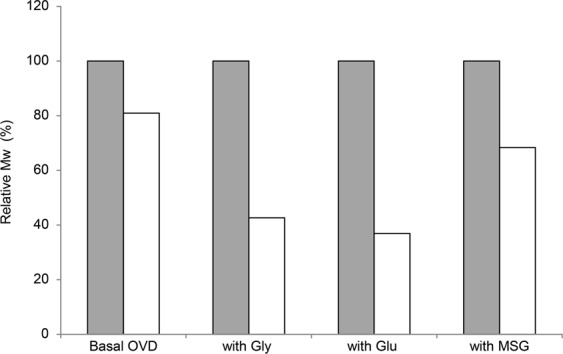
Profiles of Mw reduction of HA in OVDs with amino acids during the preservation test at 60 °C. The relative Mw (%) was calculated by using the following numerical formula: [The Mw of HA in OVDs at day 14 (Da)]/[The initial Mw of HA in OVDs (Da)] × 100. ■: Day 0; □: Day 14.

We evaluated the effect of various additives on the viscosity stability of the basal OVD. As shown in Table 2, sugar alcohols were effective in preserving the viscosity of the basal OVD; D-sorbitol was the best additive among the additives tested. The viscosity of OVD containing Gly, Glu, or MSG was extremely reduced after preservation at 60 °C for 2 weeks. Maillard products were generated in those OVDs, owing to an alteration in their color from colorless to pale brown. According to Deguine *et al*., free radicals generated in the Maillard reaction decrease the viscosity and molecular weight of HA during 1 h of incubation at 37 °C^21^. We also demonstrated depolymerization of HA in the OVD by adding amino acids in Fig. 2. Therefore, the drastic viscosity reduction by adding amino acids to the basal OVD was due to the depolymerization of HA caused by the generation of free radicals in the Maillard reaction.

The OVD containing D-sorbitol maintained viscometric property highly compared with the basal OVD, against both thermal treatment and light irradiation. It is well known that the generation of free radicals is enhanced by light irradiation or with thermal dependence. The degradation of HA caused by exposure to reactive oxygen species was reported to be inhibited in a concentration-dependent manner by D-mannitol^22,23^. Hence, the antioxidant capacity of D-sorbitol might be one possible mechanism to preserve the viscosity of the basal OVD over a series of preservation tests.

**Figure 3:**
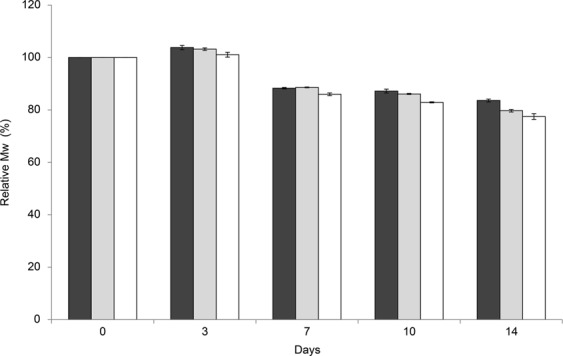
Profiles of Mw reduction of HA in OVDs with D-sorbitol during the preservation test at 60 °C. The relative Mw (%) was calculated by using the following numerical formula: [The Mw of HA in OVDs at each time (Da)]/[The initial Mw of HA in OVDs (Da)] × 100. The values represent the mean ± standard deviations (*n* = 3). ■: OVD with 1.0% D-sorbitol; ■: OVD with 0.5% D-sorbitol; □; basal OVD.

L-methionine was not able to suppress the viscosity reduction of the basal OVD in our study, though it was reported that L-methionine preserved the viscosity of HA after exposure to a myeloperoxidase-derived oxidant^24^. Therefore, other mechanisms might be responsible for the viscosity reduction of HA/CS combination products. Figure 3 shows that, surprisingly, the profiles of the molecular weight reduction of HA in both the basal OVD and OVD containing D-sorbitol were hardly different through the preservation testing. These results suggested that D-sorbitol might possess mechanisms other than antioxidant activity to preserve the viscosity of the OVD. The viscosity of the combination of HA and CS significantly increases compared with the sum of the viscosity of HA or CS alone, owing to the mutual interaction of CS and HA to form aggregates via hydrogen bonding^25,26^. D-sorbitol might cause stiffening due to the interaction between HA and CS.

Few reports demonstrate that sugar alcohols preserved the viscosity or molecular weight of HA in artificial oxidative conditions. However, there are no reports on D-sorbitol affecting the long-term stability of HA/CS combinations. To fully exploit the abilities of OVDs during surgery, it is desirable to have a sufficient viscosity. For long-term product performance, it is important to manage the preservation temperature in many HA products. Therefore, many distributors recommend that surgeons store the OVD products in a cold place like a refrigerator. Before using the refrigerated OVDs, surgeons should bring the product temperature back to room temperature, as the viscosity depends on the temperature: if the OVD is used when cold, the therapeutic efficiency and usability may be affected.

This is the first study to investigate the effects of D-sorbitol on the long-term stability of OVD viscosity. Figure 1 shows that viscosity reduction in the new composition was hardly observed during 24 months of preservation testing at 5 °C, including for the basal OVD. The transition in the viscosity of the new composition stored at 25 °C was maintained in the range of 55 to 47 Pa·s during the testing. The viscosity loss of the new composition stored at 25 °C for 24 months was very similar to the result obtained when the new composition was stored at 5 °C (87%), indicating that D-sorbitol allows the storage of OVDs at room temperature for 24 months.

Five HA products containing sugar alcohols have been launched: Synolis VA^®^ (Aptissen SA, GE, Switzerland), GO-ON^®^ matrix (Meda AB, Solna, Sweden) and Ophteis^®^ FR Pro (Rayner Ltd., WSX, United Kingdom), all of which contain 4% D-sorbitol^27–30^, and Ostenil^®^ and Visiol^®^ (TRB Chemedica Int. SA, GVA, Switzerland), both of which contain 0.5% D-mannitol^23,31^. Several reports have demonstrated that D-mannitol or D-sorbitol can improve the effectiveness of intra-articular HA products in the treatment of osteoarthritis without any serious or unexpected effects^22,28,29^. Therefore, we believe that the new combination developed in this study would aid ophthalmic surgeons in administering timely treatment.

**Figure 4:**
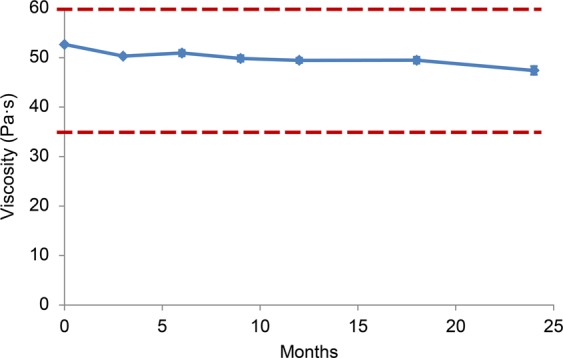
Long-term preservation stability of the manufactured product, Shellgan^®^ at 25 °C. Time-course of viscosity of Shellgan^®^ represent the mean ± standard deviations (*n* = 3). The dotted lines represent the standard value of Shellgan^®^, i.e., 35–60 Pa·s.

We demonstrated that adding D-sorbitol to a HA/CS combination can preserve its viscosity at room temperature. Thus, we developed a new OVD formulation, termed Shellgan^®^, which is the first dispersive OVD formulation that can be stored at room temperature for at least 24 months (Fig. 4). We believe that the new dispersive OVD product has the potential to be clinically effective by maximizing therapeutic efficacy concomitantly with readiness for intraoperative emergencies, because this product can maintain its viscosity for a long time even when stored at room temperature.

## Materials and Methods

### Materials

Purified HA from chicken combs and CS from shark cartilage were obtained from Seikgaku Corp. (Tokyo, Japan). D-sorbitol, glycine, L-glutamate, monosodium L-glutamate, L-methionine, D-glucose, maltose monohydrate, xylitol, and D-α-tocopherol were purchased from Wako Pure Chemical Industries Ltd. (Osaka, Japan).

### Sample preparation

To prepare basal OVD, powdered HA and CS were dissolved in phosphate-buffered saline to obtain final concentrations of 3% and 4%, respectively (Table 1, *a*). To evaluate the effect of various additives on the viscosity stability of basal OVD, we selected 9 additives, some of which showed antioxidant capacity determined by 6-hydroxy-2,5,7,8-tetramethylchroman-2-carboxylic acid (Trolox) equivalent antioxidant capacity assay (data not shown). Each additive was added to the basal OVD to obtain a final concentration of 0.5% (Table 1, *b*-*j*). To evaluate the effect of additive concentration, a sample containing 1.0% D-sorbitol was also prepared (Table 1, *k*). Every sample was filtered through a 0.22-μm membrane filter before use.

### Determination of molecular weight distribution

**Figure 5:**
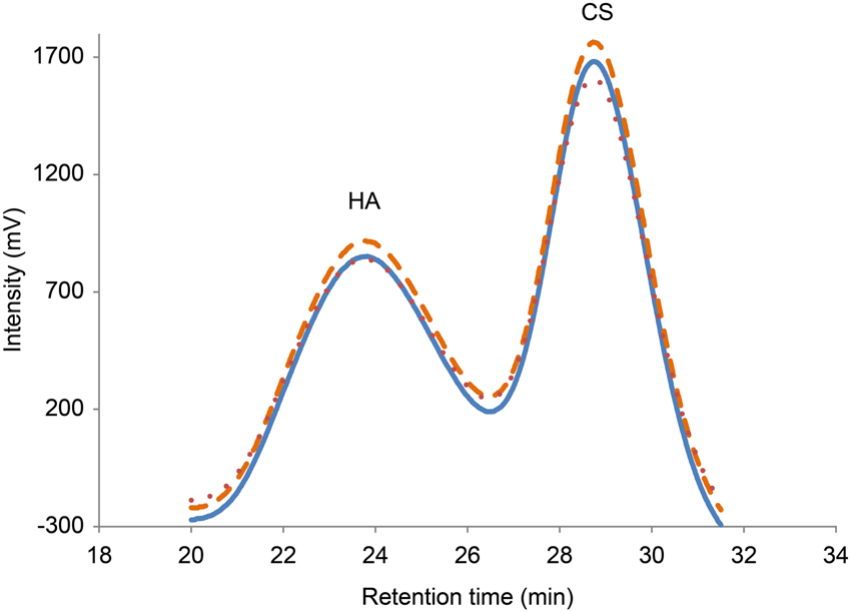
Initial molecular weight distribution of OVDs with different D-sorbitol contents. SEC-HPLC elution profiles obtained with (—) basal OVD, (---) OVD with 0.5% D-sorbitol, and (•••) OVD with 1.0% D-sorbitol. The peaks detected at 20–26 min and 26–31 min correspond to HA and CS, respectively.

The molecular weight distribution of sample was determined by high-performance liquid chromatography (LC Prominence; Shimadzu Co., Ltd., Kyoto, Japan) using a size exclusion column (OHpak SB-806M HQ; Shodex, Co., Ltd., Tokyo, Japan) and a reflective index detector (RID-10A; Shimadzu Co., Ltd.). The flow rate was 0.3 mL min^−1^ of 0.5 M NaCl at 35 °C. Each sample was diluted 300 times by the eluent and aliquots (100 μL) before injection. The initial molecular weight distribution and viscosity of each OVD were independent of additives. The elution profiles of the basal OVD and OVD containing D-sorbitol are shown in Fig. 5. Relative weight-average molecular weight (Mw) was calculated by using 6 Pullulan standards for molecular weight (Shodex, Co., Ltd.).

### Preservation stability testing at 60 °C

Each sample was stored for 14 days in dark at 60 °C. At each measuring point, i.e., after 0, 3, 7, 10, and 14 days, the viscosity of each sample was determined by using a rotational viscometer (TVE-22H; Toki Sangyo Co., Ltd., Tokyo, Japan) at a shear rate of 2 s^-1^ at 25 °C. Residual viscosity (%) was calculated by the following numerical formula: [The viscosity at each evaluation (Pa·s)]/[The viscosity of sample at the initial time (Pa·s)] × 100.

### Thermal stability testing

Samples with different D-sorbitol concentrations were autoclaved at 121 °C for 5 min. The residual viscosity of all the samples were determined by the method described above, both before and after autoclaving.

### Light stability testing

Plastic syringes (Schott AG, MAI, Germany) filled with samples were irradiated in photostability chambers (LT-120 D3J; Nagano Science Co., Ltd., Osaka, Japan) with 2000 lx h^−1^ light at 25 °C for 25 days. As a negative control, syringes shielded by aluminum foil were used. Viscosities of each sample before and after testing were measured by a rotational viscometer under the same conditions as mentioned above, and residual viscosity was calculated according to the above-mentioned formula.

### Long-term shelf-life testing at 5 °C or 25 °C

Plastic syringes filled with the sample containing 0.5% D-sorbitol was stored in dark for 24 months at 5 °C or 25 °C and 60% humidity. At each measuring point, i.e., after 0, 3, 6, 9, 12, 18 and 24 months, the viscosity of each sample was determined by the method described above.

## Data Availability

All data generated or analysed during this study are included in this published article.
